# Correlation between T2-weighted CMR and Sestamibi-SPECT in acute myocardial infarction and acute coronary occlusion

**DOI:** 10.1186/1532-429X-11-S1-P7

**Published:** 2009-01-28

**Authors:** Martin Hadamitzky, Nadine Kirchhartz, Eva Hendrich, Stefan Martinoff, Albert Schömig

**Affiliations:** Deutsches Herzzentrum München, Munich, Germany

**Keywords:** Single Photon Emission Compute Tomography, Percutaneous Coronary Intervention, Acute Myocardial Infarction, Perfusion Defect, Defect Size

## Background

Being a consistent finding in acute myocardial infarction, T2 weighted MRI imaging has recently been proposed as a marker for the area not perfused because of the underlying coronary occlusion. But data comparing T2 imaging with the gold standard for perfusion, acute Sestamibi SPECT, is very limited. In particular the interval between interruption of perfusion and onset of the cell edema in humans is unknown.

## Methods

We therefore performed in 38 patients with acute myocardial infarction (interval between start of symptoms and intervention below 24 h) and 10 patients with acute coronary occlusion as a complication during percutaneous coronary intervention both a myocardial single photon emission computed tomography (SPECT) and a cardiac MRI with fat suppressed T2-weighted turbo spin echo sequences. For SPECT 99mTc-Sestamibi applied before coronary revascularization, the measurement was done immediately after the intervention; the MRI was done 2 to 6 days after intervention. The area of increased T2 signal was quantified automatically using a cutoff of mean plus 2 standard deviations of the signal intensity in a remote myocardial region. In SPECT the area of risk was defined as area of intensity below 50% of maximum. Both values were expressed as fraction of left ventricular myocardial volume.

## Results

**Figure 1:**
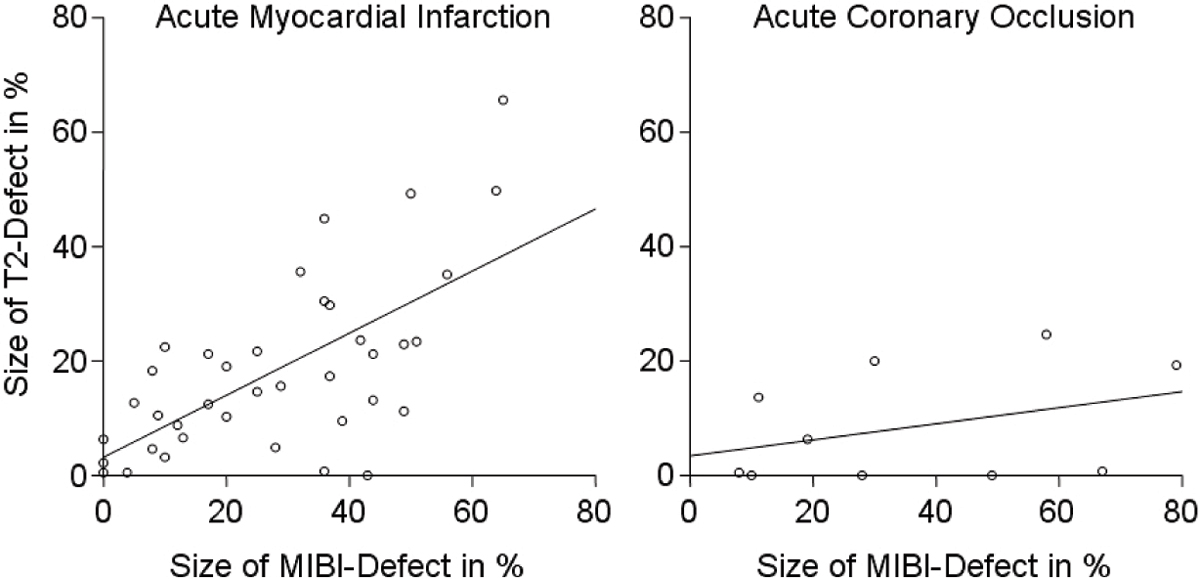
Figure 1

In clinical AMI the defect size of T2 weighted MRI ranged between 0% and 66% (18% mean), the defect size of SPECT between 0% and 65% (28% mean). There was a highly significant correlation between the two measurements with a correlation coefficient of 0.65 as depicted by the left image in Figure 1.

In acute coronary occlusion the defect size of T2 weighted MRI (9%) was only one fourth of the defect size of SPECT (36%). There was no significant correlation between the two measurements (r = 0.14, p = 0.31, see also right image below).

## Conclusion

T2-weighted CMR shows a good correlation to acute Sestamibi SPECT in depicting perfusion defects in clinical acute myocardial infarction, but it clearly underestimates short-lived perfusion defects as seen in acute coronary occlusions during PCI.

